# Associations of MicroRNAs, Angiogenesis-Regulating Factors and CFH Y402H Polymorphism—An Attempt to Search for Systemic Biomarkers in Age-Related Macular Degeneration

**DOI:** 10.3390/ijms20225750

**Published:** 2019-11-15

**Authors:** Zofia Ulańczyk, Anna Sobuś, Karolina Łuczkowska, Aleksandra Grabowicz, Katarzyna Mozolewska-Piotrowska, Krzysztof Safranow, Miłosz Piotr Kawa, Andrzej Pałucha, Mariusz Krawczyk, Piotr Sikora, Ewa Matczyńska, Bogusław Machaliński, Anna Machalińska

**Affiliations:** 1 Department of General Pathology, Pomeranian Medical University, 70-111 Szczecin, Poland; z.litwinska@gmail.com (Z.U.);; 2 First Department of Ophthalmology, Pomeranian Medical University, 70-111 Szczecin, Poland; 3 Department of Biochemistry and Medical Chemistry, Pomeranian Medical University, 70-111 Szczecin, Poland; 4 Genomed SA, 02-971 Warsaw, Poland

**Keywords:** AMD, miRNA, CFH, growth factors, angiogenesis

## Abstract

Age-related macular degeneration (AMD) remains the leading cause of blindness in elderly people, but the pathophysiology of this disease is still largely unknown. We investigated the systemic expression of angiogenesis-regulating growth factors and selected miRNAs known to regulate angiogenesis in AMD patients. We also focused on possible correlations of their expression with the presence of CFH Y402H or ARMS A69S risk variants. A total of 354 AMD patients and 121 controls were enrolled in this study. The levels of angiogenesis-regulating factors were analyzed in plasma samples using Luminex technology. The expression of selected miRNAs was analyzed in peripheral blood plasma using real-time qPCR. The genetic analysis was performed with an Illumina NextSeq500 system. AMD was an independent factor associated with lower levels of angiogenin (β = −0.29, *p* < 0.001), endostatin (β = −0.18, *p* < 0.001), FGF-basic (β = −0.18, *p* < 0.001), PlGF (β = −0.24, *p* < 0.001), miRNA-21-3p (β = −0.13, *p* = 0.01) and miRNA-155-5p (β = −0.16, *p* = 0.002); and with higher levels of FGF-acidic (β = 0.11, *p* = 0.03), miRNA-23a-3p (β = 0.17, *p* < 0.001), miRNA-126-5p (β = 0.13, *p* = 0.009), miRNA-16-5p (β = 0.40, *p* < 0.001), miRNA-17-3p (β = 0.13, *p* = 0.01), miRNA-17-5p (β = 0.17, *p* < 0.001), miRNA-223-3p (β = 0.15, *p* = 0.004), and miRNA-93 (β = 0.11, *p* = 0.04). The expression of analyzed miRNA molecules significantly correlated with the levels of tested angiogenesis-regulating factors and clinical parameters in AMD patients, whereas such correlations were not observed in controls. We also found an association between the CFH Y402H polymorphism and miRNA profiles, whereby TT homozygotes showed evidently higher expression of miRNA-16-5p than CC homozygotes or TC heterozygotes (*p* = 0.0007). Our results suggest that the balance between systemic pro- and anti-angiogenic factors and miRNAs is vital in multifactorial AMD pathogenesis.

## 1. Introduction

Age-related macular degeneration (AMD) is a common ocular condition characterized by progressive visual impairment [1]. AMD currently remains the leading cause of blindness in the elderly population, which profoundly disrupts quality of life [2,3].

AMD has two main forms: exudative/wet and non-exudative/dry [4]. The prevalence of dry AMD is higher than that of wet AMD, comprising 80–85% of all cases, and dry AMD is associated with retinal pigment epithelium (RPE) and photoreceptor degeneration [5]. The advanced form of AMD, the so-called ‘wet’ AMD, is associated with choroidal neovascularization (CNV) and results in rapid visual acuity loss, accounting for 90% of all clinical cases with loss of sight [6,7]. The new vessels that are formed during neovascularization are largely malformed and account for improper vascular integrity [8]. This leads to undesirable blood and fluid leakage within the disrupted tissue, which stimulates inflammation and scar formation and results in retinal damage [9]. This damage to the retina accounts for central vision loss and, if not treated, eventually blindness [6,10]. Unfortunately, the complex pathogenesis of this disease is not fully understood, and currently, there is no established biomarker for early AMD detection.

AMD has a strong genetic component; linkage studies have found two major AMD loci mapped on chromosomes 1q32 and 10q26 [11]. The first locus harbors complement factor H (CFH), whose polymorphism (Y402H) is considered the “risk variant”, accounting for a population attributable risk fraction for early and late AMD of approximately 10% and 53% [12], respectively. The ARMS A69S polymorphism at 10q26 is thought to cause misfolding of the mitochondrial-associated protein encoded in this region [13]. While the pathogenetic role of CFH Y402H is through the complement system, the exact role of ARMS A69S in AMD susceptibility remains unclear.

Although the exact causes of AMD remain an active research area, it is known that angiogenesis and vascular imbalance play a crucial role in this disease, with vascular endothelial growth factor (VEGF), a potent pro-angiogenic factor, likely being a pivotal point [2,10]. Several eye cells produce VEGF, including RPE cells, endothelial cells, glial cells, pericytes, Muller cells, and ganglion cells [14]. Apart from blood vessel growth stimulation, VEGF also stimulates endothelial cells to produce matrix metalloproteinases (MMPs), a family of enzymes that proteolytically degrade various components of the extracellular matrix and thus allow new vessels to grow [15]. A considerable number of studies have shown that inhibition of VEGF production can suppress CNV in the eye, and anti-VEGF therapeutics have been developed [16]. Although widely used, VEGF inhibitors show some detrimental side effects (including stroke) [17] and are apparently ineffective in a subset of AMD patients [18]. Recently, Keir et al. provided an interesting link between VEGF and CFH, showing that VEGF inhibition decreases local CFH complement regulators in the eye through reduced VEGFR2/PKC-α/CREB signaling [19]. In that study, RPE cells carrying disease-associated CFH genetic variants had more alternative complement pathway deposits than controls, and these deposits were increased by VEGF antagonism. Apart from VEGF, other growth factors regulate angiogenesis and could play a role in AMD, including platelet-derived growth factor (PDGF), fibroblast growth factors (FGFs), pigment epithelium-derived growth factor (PEDF), and angiopoietins (ANGPTs) [6,20]. Recently, the presence of several angiogenesis inhibitors—including thrombospondin-1 (TSP1), PEDF, endostatin, and angiostatin—has been reported in the eye environment [14]. Therefore, it seems that a balanced production of negative and positive angiogenesis-regulating growth factors is essential for achieving ocular vascular homeostasis. Various stimuli—e.g., hypoxia, oxidative stress, ischemia, and inflammation (which all accumulate with age)—can influence the production of those growth factors and skew this desirable balance, leading to AMD development or progression [6].

A major role in governing various pathological processes that contribute to AMD pathogenesis, including angiogenesis, has recently been attributed to microRNAs (miRNAs, miRs) [21]. These small, noncoding RNAs of approximately 20 nucleotides are potent gene expression regulators that have been found in a variety of body fluids, i.e., blood, saliva, and urine [22,23]. Changes in miRNA expression have been implicated in common human disorders including AMD, and the potential use of miRNAs as biomarkers seems encouraging [21,24,25]. Several researchers have shown that certain miRNAs are abundant in the vasculature and play an important role in angiogenesis regulation [3,26]. Numerous miRNAs—including the miRNA-17-92 cluster and also miRNA-21, miRNA-93, miRNA-126, miRNA-221, and others—have also been linked specifically with ocular angiogenic processes [3,27,28]. In fact, it seems that miRNAs are also closely associated with *CFH* risk variants in AMD. Interestingly, in a study by Lukiw et al., four miRNAs (miRNA-9, miRNA-125b, miRNA-146a, and miRNA-155) were upregulated in whole retina samples from AMD patients compared to healthy controls [29]; all of those miRNAs have been shown to specifically bind to the 3′UTR of the *CFH* gene, thus possibly being major regulators of its expression. It seems that the involvement of miRNAs in angiogenesis and CFH expression in AMD is substantial, making miRNAs presumably major governing forces in AMD pathogenesis.

In the present study, we aimed to explore the expression of systemic angiogenesis-regulating growth factors and selected peripheral blood plasma miRNAs that regulate angiogenesis in AMD patients. We also focused on possible correlations of their expression with the presence of CFH Y402H or ARMS A69S risk variants in our patients.

## 2. Results

### 2.1. Characteristics of the Study Subjects

We enrolled 354 patients with AMD (175 dry AMD, 179 wet AMD) and 121 healthy controls in the study. The clinical characteristics of the patients and controls are summarized in Table 1. We analyzed vascular-related risk factors in the study groups because epidemiological data collected so far show that AMD is associated with atherosclerosis. The AMD and control groups did not present with significant differences in age or well-known atherosclerotic risk factors, namely, hypertension, history of ischemic heart disease, cardiac infarction, cerebral stroke, peripheral artery disease, and aortic aneurysm. The proportion of past smokers and the number of smoking pack-years was significantly higher in the AMD group than in controls (*p*  =  0.0004 and *p* < 0.0001, respectively).

### 2.2. Levels of Angiogenesis-Regulating Factors

To assess the systemic levels of angiogenesis-related factors, we chose 10 for analysis in plasma: angiogenin, angiopoietin-1, endostatin, FGF-basic, FGF-acidic, PDGF-AA, PlGF, thrombospondin-2, VEGF, and VEGF-D. The AMD group presented with lower levels of four tested cytokines (angiogenin, endostatin, FGF-basic, PlGF) in comparison with the control group (Table 2(a)). Multivariate analysis of patients and controls adjusted for age, sex, and smoking status (pack-years) revealed that AMD was an independent factor associated with lower levels of angiogenin (β = −0.29, *p* < 0.001), endostatin (β = −0.18, *p* < 0.001), FGF-basic (β = −0.18, *p* < 0.001), and PlGF (β = −0.24, *p* < 0.001) and with higher concentration of FGF-acidic (β = 0.11, *p* = 0.03). AMD subtype analysis did not reveal any statistically significant differences in the concentrations of the tested factors (Table 2(b)).

### 2.3. Plasma miRNA Expression Profiles

As a next part of our study we analysed the expression of several miRNAs in AMD and control patients’ blood plasma samples using qRT-PCR. For this part, we chose 14 miRNAs (miRNA-9-5p, -16-5p, -17-3p, -17-5p, -21-3p, -23a-3p, -30b, -93, -126-5p, -146a, -150-5p, -155-5p, -191-5p, and -223-3p) targeting angiogenic, inflammatory, and cell survival processes in response to oxidative stress with documented link to AMD pathogenesis [3,21,30]. Seven analyzed miRNAs (miRNA-16-5p, miRNA-17-3p, miRNA-17-5p, miRNA-23a-3p, miRNA-126-5p, miRNA-146a, and miRNA-223-3p) showed higher expression and three (miRNA-21-3p, miRNA-155-5p, and miRNA-191-5p) showed lower expression in AMD patients in comparison with controls (Table 3(a)). In multivariate analysis, AMD was an independent factor associated with higher expression of miRNA-23a-3p (β = 0.17, *p* < 0.001), miRNA-126-5p (β = 0.13, *p* = 0.009), miRNA-16-5p (β = 0.40, *p* < 0.001), miRNA-17-3p (β = 0.13, *p* = 0.01), miRNA-17-5p (β = 0.17, *p* < 0.001), miRNA-223-3p (β = 0.15, *p* = 0.004), and miRNA-93 (β = 0.11, *p* = 0.04); and lower expression of miRNA-21-3p (β = −0.13, *p* = 0.01) and miRNA-155-5p (β = −0.16, *p* = 0.002). Wet and dry AMD patients also showed slight differences between their plasma miRNA profiles (Table 3(b)).

Next, we aimed to investigate if and how severity of underlying disease could impact miRNA profile. In order to do that, we studied the association between miRNAs expression and selected clinical parameters. We observed significant relationship between miRNA profile and retinal and choroidal parameters: positive correlations were observed for central choroidal thickness values and miRNA-93 expression (Rs = +0.119, *p* = 0.03) and thickness of the central retina and the expression of miRNA-16-5p (Rs = +0.159, *p* = 0.004) and miRNA-223-3p (Rs = +0.129, *p* = 0.02). A negative correlation was observed between visual acuity and miRNA-23a-3p (Rs = −0.141, *p* = 0.01). Remarkably, no such correlations were found in the control group.

### 2.4. miRNA Correlations

To more accurately characterize the role of the analyzed miRNA profiles in AMD patients, we evaluated the association between the expression of miRNA molecules and levels of angiogenesis-regulating factors in plasma. We aimed to investigate whether certain miRNAs are linked with angiogenic factors and whether these correlations are specific for AMD patients or wet/dry AMD subtypes. In general, we found that the expression of analyzed miRNA molecules significantly correlated with the levels of tested angiogenesis-regulating factors in AMD patients, whereas such correlations were not observed in controls (Table 4 and Table 5). The only miRNA whose statistically significant correlations with angiogenesis-regulating factors were only negative was miRNA-17-3p. In fact, a negative correlation between PlGF levels and miRNA-17-3p expression (Rs = −0.147) corresponded with a significant reduction in PlGF levels and increase in the expression of this miRNA observed in AMD patients. The complementary strand of this miRNA, miRNA-17-5p, showed a negative correlation with FGF-acidic, just as miRNA-17-3p did, and a unique positive correlation with endostatin. Only two other miRNAs correlated positively with endostatin (miRNA-93 and miRNA-150-5p), and neither of them showed a difference in expression between AMD patients and controls. Interestingly, the miRNAs that correlated negatively with endostatin showed elevated expression in the plasma of AMD patients (miRNA-23a-3p, miRNA-146a, and miRNA-223-3p). A similar situation was observed for angiogenin, whose levels were lower in the AMD group and correlated negatively with the same three miRNAs (miRNA-23a-3p, miRNA-146a, and miRNA-223-3p), which were elevated in the AMD group. In the case of miRNA-223-3p, this correlation also occurred in the controls; however, the remaining miRNA correlations were unique to the AMD group. This could suggest that these miRNAs are negative regulators of angiogenin and endostatin in AMD patients. Overall, the observed diversity of miRNA-angiogenesis factor correlations reflects the vast variety of biological processes that these miRNAs regulate. 

### 2.5. Genotypes and miRNAs

We analyzed associations between plasma miRNAs and genotypes with regard to polymorphisms in genes previously linked with AMD: CFH Y402H and ARMS A69S. The only statistically significant associations were observed for CFH Y402H (Table 6). Two miRNAs showed significantly lower expression in patients with the C allele (miRNA-16-5p and miRNA-17-5p); however, when correction for multiple testing was applied, only the association of miRNA-16-5p with the C allele remained statistically significant. TT homozygotes showed evidently higher expression of miRNA-16-5p (median = 16.3) than CC homozygotes (median = 12.25) or TC heterozygotes (median = 11.70) (Table 6).

## 3. Discussion

AMD is a multifactorial disease of the central retina involving a range of genetic and environmental influences. All of the changes associated with ageing in the outer retina weaken the nutrition of photoreceptors and RPE and create a favorable environment for the development of choroidal neovascularization (CNV) [31]. The shift in balance between pro- and anti-angiogenic factors is thought to play a key role in CNV development [9]. This balance could be regulated by miRNAs, which have taken prominent positions in our understanding of the control of various biological processes, including angiogenesis [21]. Thus, in the present study, we aimed to explore the pathological angiogenesis in AMD by studying the expression of systemic angiogenesis-regulating growth factors, selected genetic predispositions and peripheral blood plasma miRNAs.

First, we assessed the systemic levels of angiogenesis regulators in peripheral blood plasma and found that the central mediator of angiogenesis, VEGF, did not show differences between the tested groups, whereas four other factors presented with lower levels in the AMD group: angiogenin, endostatin, FGF-basic, and PlGF. Since anti-VEGF agents are widely used in the treatment of neovascular or exudative AMD, it is not surprising that VEGF plasma levels are extensively studied as a measure of response to therapy [32,33]. Similar to Carneiro et al. [34], we could not confirm that patients with exudative AMD had higher levels of VEGF plasma compared to age-matched controls, as previously reported by Tsai et al. [35]. This confirms the importance of patient selection: whereas Tsai et al. reported data from patients with an Asian background, the patients presented here and in the Carneiro et al. studies are Caucasian. VEGF signaling is downregulated by endostatin, which targets thrombin receptors, Stats, and HIF-1 and simultaneously upregulates antiangiogenic genes (vasostatin and kininogen) in human microvascular endothelial cells (EC) [10,36,37]. In 2006, Tatar et al. showed that endostatin is expressed in human choroidal neovascular membranes (CNV) secondary to age-related macular degeneration [38]. Intravitreal or intravenous delivery of endostatin by viral vectors was also shown to inhibit diabetic retinopathy and CNV in experimental studies [39,40]. It is possible that endostatin delivery could be beneficial to our AMD patients, who presented with lower levels of endostatin in comparison to controls. However, we found no differences in endostatin levels between dry and wet AMD types, which requires further study. In contrast to anti-angiogenic endostatin, angiogenin, FGF-basic, and PlGF had pro-angiogenic properties and decreased levels in our AMD patients. These growth factors have been implicated in various diseases, including cancer, but little is known about their expression and role in ocular neovascular disorders [41,42,43,44]. Angiogenin, FGF-basic and PlGF levels were previously shown to be elevated in the vitreous of proliferative diabetic retinopathy patients, CNV membranes from patients with AMD and/or mouse models of laser-induced CNV [45,46]. Targeted disruption of *ANG*, *FGF-2*, or *PlGF* genes gave discrepant results in reducing experimental CNV [47,48], which supports the notion that a single factor is not sufficient to induce CNV in the absence of additional stimuli. The reduction of systemic FGF-basic and PlGF observed in our study are particularly interesting, since both factors have been shown to act in cooperation with VEGF to accelerate neovascularization and dual blockade of VEGF with either FGF-basic or PlGF has shown potent anti-angiogenic effects [49,50,51,52,53]. Our results correspond with the ongoing research on the role of PlGF and FGF-basic in coordination with VEGF in CNV progression. However, our finding on PlGF downregulation stands in contrary to recent results by Ioanna et al. who have shown elevated plasma levels of PlGF in wet AMD [54]. On the other hand, Zhou et al. have recently suggested [55], based on their study on neovascular glaucoma, local PlGF and VEGF production directly in the eye as opposed to transudation of these factors from blood into the vitreous. Although the decrease in the levels of pro-angiogenic factors in our study might be a result of ongoing AMD processes in the eye, with quick turnover of selected angiogenesis-stimulating factors, rather than a cause of those disturbances, this requires further in-depth studies.

The association between miRNA dysregulation and AMD pathogenesis is becoming evident; both vitreous miRNA expression profiles and miRNA serum/plasma signatures characteristics of AMD patients are being researched [56,57]. Our study provides further evidence that miRNA assessment in peripheral blood plasma is feasible to distinguish between wet and dry AMD types. In the present study, four miRNAs (miRNA-16-5p, miRNA-23a-3p, miRNA-30b, and miRNA-191-5p) were differentially expressed between those groups. The number of correlations found in our study between miRNAs and angiogenesis-regulating factors confirms the wide involvement of miRNAs in regulating this process. Not only are miRNAs key regulators of angiogenesis (a group of miRNAs called ‘angiomiRs’, either pro-angiomiRs or anti-angiomiRs), but they can be regulated by pro- or anti-angiogenic stimuli, including hypoxia, or can have a dual function in angiogenesis [58]. The miR-17-92 cluster is an important regulator of the angiogenic switch [59], and we tested the two strands of one of its family members, miRNA-17. This miRNA is predicted to target the *HIF-1α* and *VEGF-A* genes [60], although in our study we only found significant correlations with VEGF-D concentration. This cluster is highly expressed in ECs and is upregulated by ischemia [61]. It shows vast anti-angiogenic action; thus, it is possible that an increase in miRNA-17 in our AMD group is a systemic attempt to rescue the ocular tissue from increased angiogenesis. Similarly, miRNA-21-3p is induced by hypoxia and has been shown to inhibit neovascularization [30]. However, there are some discrepancies regarding miRNA-21-3p function, as some researchers have suggested that miRNA-21 promotes the tube-forming capacity of endothelial cells [62], and some studies have argued that, in fact, miRNA-21 reduces EC the proliferation, migration, and ability to form tubes of these cells [63]. Our results of decreased miRNA-21 levels in AMD patients are in concordance with the report by Ertekin et al. [64], where the authors studied nAMD patients in comparison to controls. Contradictory functions have also been described for miRNA-126, suggesting that it probably plays dual roles and cell-dependent functions in regulating angiogenesis [65]. Our results could also be interpreted in two ways: either miRNA-126 promotes angiogenesis, which is more profound in the AMD group; hence, the increased expression of miRNA-126, or it is a protective mechanism, similar to the miRNA-17-92 cluster. Recently, Wang et al. reported that miRNA-126 is profoundly decreased in a laser-induced CNV model [66]. MiR-126 levels were also decreased in diabetic retinopathy or retinopathy of prematurity cases [67,68]; however, none of these studies were performed on human subjects. MiRNA-146a and miRNA-155 are equal candidates for AMD biomarkers since their recognition sites in the *CFH* mRNA 3′UTR overlap, and they showed similar expression patterns in mouse models and ocular tissue [29]. In our study, these two miRNAs correlated with similar angiogenesis-regulating factors, but their expression differed (miRNA-146a increased and miRNA-155 decreased) in the AMD group. MiR-146a has been linked to progressive, age-related, inflammatory neurodegenerative disorders [69]. MiR-146a has been hypothesized to modulate innate immune responses, inflammation, and the microglial activation state; however, it remains to be established whether the induction of miR-146a is protective or detrimental to the cell [70]. Several studies have shown that miR-146a upregulation in stressed human neural cells results in the downregulation of CFH, a major repressor of the innate immune and inflammatory responses and key player in AMD pathogenesis [71]. Contrary to our findings, miRNA-155 is upregulated during AMD [72]; several studies have shown its upregulation in AMD retina and macula, in oxygen-induced retinopathy (OIR) in mouse retina, and in rat retinas after light-induced retinal degeneration [27]. In fact, miRNA-155 deficiency does not seem to bring any protective effects. When Yan et al. subjected miR-155 knockout mice to OIR, it induced rapid revascularization and prevented the development of aberrant neovascularization [73]. Our finding of miRNA-16-5p and CFH Y402H association has not been previously described in the literature. MiRNA-16 is one of the first miRNAs to be linked to human malignancies and has several important targets: the anti-apoptotic gene Bcl-2 (B-cell lymphoma 2); numerous genes involved in the G1-S transition, such as cyclin D1, cyclin D3, cyclin E1, and CDK6 (cyclin-dependent kinase 6); and genes involved in the Wnt signaling pathway, such as WNT3A (wingless-type MMTV integration site family, member 3A) [74]. In our study, the C allele in CFH Y402H, which is considered a risk allele in AMD [75], was linked with lower expression of miRNA-16. This might be interpreted as insufficient cell cycle control by low miRNA-16 expression in patients harboring the risk allele (although the expression is still evidently higher than that in controls); however, this novel hypothesis requires further investigation.

The role of miRNA in human physiology and pathology, including age-related macular degeneration, seems crucial. They could serve as potential biomarkers, helpful in diagnosing the disease and monitoring treatment effect, but also being a starting point for development of personalized therapy [76,77,78,79]. The potential application of the research includes differentiating the etiopathogenetic pathways leading to various forms of AMD as well as the development of type-specific panel of AMD risk variables. The ongoing research on miRNA involvement in AMD will provide not only promising therapeutic options, but also enable better understanding of this disease mechanisms.

## 4. Conclusions

AMD is a complex disease of the retina, and its pathophysiology remains unclear. Our study provides evidence that miRNAs could serve as potential biomarkers for distinguishing between wet and dry AMD. Our novel finding of lower miRNA-16-5p expression in CFH risk allele carriers provides a presumed link between miRNA expression profiles and AMD pathogenetics; however, this needs further clarification. The dual role of some miRNAs in angiogenesis makes it challenging to draw conclusions about their exact actions; however, it seems that a proper balance between systemic pro- and anti-angiogenic factors and miRNAs is vital for the maintenance of retinal homeostasis. It remains to be further elucidated whether the systemic response observed in our in vivo study reflects ongoing degenerative processes or whether it is a protective attempt to diminish excessive retinal neovascularization.

## 5. Materials and Methods

### 5.1. Study Group Characteristics

We recruited 354 patients with newly diagnosed AMD from the outpatient population of the First Department of Ophthalmology of Pomeranian Medical University in Szczecin, Poland for participation in this study. As a control group, 121 age-matched participants with no symptoms or signs of macular degeneration (defined as the absence of drusen, pigmentary abnormalities, or neovascularization) were included in the study. All patients from AMD and control group were subjected to a complete ophthalmic examination including optical coherence tomography (OCT) analysis (Spectralis, Heidelberg Engineering, Heidelberg, Germany), visual acuity (VA), and intraocular pressure measurements and dilated fundus examination using slit-lamp biomicroscopy. Snellen letter chart was used to measure VA, which was later transformed to LogMAR (Logarithm of the minimum angle of resolution) for statistical analyses.

From all enrolled AMD subjects, 179 patients had a clinical diagnosis of wet AMD, with newly diagnosed CNV (subretinal neovascular membrane, subretinal hemorrhage, serous or hemorrhagic retinal pigment epithelium detachment, fibrous scar). The remaining 175 AMD patients were diagnosed with dry AMD, with visible alterations in RPE (geographic retinal atrophy and macular drusen). If different disease stages were diagnosed in each eye, the severity of changes in the worse eye were a deciding factor for assigning a patient to a wet/dry group. Patients presenting with significant chronic systemic conditions (collagen/neoplastic disease, diabetes mellitus, renal failure, hepatic dysfunction) or any evidence of retinal disease except AMD (in AMD groups)—i.e., glaucoma or intraocular inflammatory diseases—were excluded from the study.

In all patients enrolled in the study, we assessed waist circumference (cm), waist/hip ratio (WHR), and body mass index (BMI) [weight (kg)/height (m)^2^]. All data regarding medical history, working conditions, smoking, and current drug use were collected from laboratory data, pathology tests, and other information. The reported average number of cigarettes smoked per day and the number of years of smoking were used for calculating cumulative pack-years. Before ophthalmic examination, in all subjects we measured the actual arterial blood pressure (BP) using a noninvasive blood pressure system with a manual aneroid manometer; three measurements obtained with 5-min resting intervals gave the mean result. From the obtained BP data, the systemic mean arterial pressure (MAP) was calculated as follows: MAP = diastolic BP + 1/3 (systolic BP-diastolic BP) mmHg.

The study adhered to the tenets of the Declaration of Helsinki, and approval was obtained from the Local Research Ethics Committee. Each patient enrolled in this study provided written informed consent for his or her involvement.

### 5.2. Blood Sample Collection

From each patient enrolled in this study, a sample of venous blood (~7.5 mL) was collected in EDTA tubes, then centrifuged (2000 rpm, 4 °C, 10 min) to collect plasma, which was stored at −20 °C to −80 °C until further assayed.

### 5.3. DNA Isolation

First, red blood cells were lysed using ammonium chloride-based lysing buffer (BD Biosciences, Franklin Lakes, NJ, USA). Nucleated cells were then counted and subsequently DNA isolation was performed with total DNA isolation kit (Macherey-Nagel, Düren, Germany), according to manufacturer’s protocol.

### 5.4. Genetic Analysis

#### 5.4.1. Exon Capturing with Molecular Inversion Probes (MIPs)

Molecular Inversion Probes (MIPs) were designed for two AMD-related genes (CFH and ARMS2) with the MIPgen program [80] that has the ability to integrate unique molecular identifiers sequences (UMI) to the designed probes, enabling reliable removal of PCR duplicates in downstream analyses. Additional 3′ and 5′ flanking sequences were added to the probes to facilitate the PCR amplification. The probes were synthesized by CustomArray Inc. (Bothell, WA, USA) and subsequently amplified to generate active probes at higher amounts for high-throughput applications. MIP amplification and capturing of targeted exons with MIPs was carried out according to the protocol published by the Sanger Institute with slight modifications (manuscript in preparation). The final library was purified with Agencourt AMPure XP (Beckman Coulter, Brea, CA, USA), quantified with Picogreen (ThermoFisher), pooled and run on the 2100 Bioanalyzer (Agilent, Santa Clara, CA, USA). The final library was sequenced on NextSeq500 (Illumina, San Diego, CA, USA) in the PE150 read mode.

#### 5.4.2. Bioinformatic Analysis

Sequencing adapters were trimmed with Cutadapt v1.14 [81] and resulting sequences were aligned to the human reference genome (hg19 version) using Burrows-Wheeler Aligner (BWA v0.7.10) [82]. Deduplication of read pairs with same UMIs, along with primer sequences removal, was performed with custom in-house scripts and verified by manual inspection. Variant calling was based on the GATK package v3.5 [83]. Both HaplotypeCaller (HC) and UnifiedGenotyper (UG) were used, preceded by best practice indel realignment and base recalibration. Variants obtained from the HC joint calling GVCF mode were subjected to additional quality filtering with the following criteria: the genotype for particular locus was possible to ascertain in at least 94% of samples and 80% of genotypes for particular variant were called with minimal coverage of 10x. The final set of variants was annotated with Ensembl Variant Effect Predictor (VEP v92.0) [84] and formatted conveniently for the statistical analysis.

### 5.5. Luminex Assay

Angiogenin, FGF-basic, endostatin, FGF-acidic, PDGF-aa, PIGF, thrombospondin-2, VEGF-d, angiopoietin-1, and VEGF concentrations were measured in plasma by multiplex fluorescent bead-based immunoassays (Luminex Corporation, Austin, TX, USA) using commercial R&D Systems Human Angiogenesis A Premixed Mag Luminex Performance Assay (R&D Systems, Minneapolis, MN, USA), according to manufacturer’s protocol. In brief, 100 μL of blank, standards and samples were added together with Microparticle Cocktail to the plate and then incubated for 2 h in the dark at room temperature (RT) on horizontal orbital microplate shaker set at 800rpm. Next, the wells were washed three times with 100 µL of wash buffer using a hand-held magnet. The plate was incubated with agitation at RT for 60 min in the dark with biotin-antibody cocktail (50 µL). After washing, Streptavidin–PE (50 µL per well) was added and the plate was incubated for 30 min in the dark on a plate shaker. After next washing step, the microspheres were resuspended in 100 µL wash buffer and shaken for 2 min at RT. Luminex 200 analyzer was used for reading and analyzing the plate and angiogenic factors concentrations were determined from seven different standard curves showing median fluorescence intensity vs. protein concentration.

### 5.6. MiRNA Analysis

Circulating miRNA was isolated from 300 µL of plasma using NucleoSpin miRNA Plasma (Macherey-Nagel, Germany) following the manufacturer’s protocol. Before isolation 25 fm of synthetic miR-39 from *C. elegans* miRNA (Institute of Biochemistry and Biophysics, Polish Academy of Sciences) was added to each sample. qScript microRNA cDNA Synthesis Kit (Quantabio, MA, USA) was used for first strand cDNA synthesis; we used 4 µL of each sample for cDNA synthesis. miRNA expression was assessed using qPCR performed with Bio-Rad CFX96 Real-Time Detection System (Bio-Rad, CA, USA). Reaction solution was as follows: 1 µL of cDNA sample, iQ SYBR Green Supermix (Bio-Rad, CA, USA), Universal Primer from qScript micrRNA Synthesis Kit, specific forward primer for analyzed miRNA. Quantification of the target miRNA expression value was expressed as 2∆Ct and NormFinder algorithm was used to find the best reference gene. Ct results were normalized to spike-in C. elegans miRNA and miR-223-5p.

### 5.7. Statistical Analysis

Prior to further analysis, quantitative parameters measured in both eyes were averaged. We used nonparametric Mann–Whitney test to compare values between groups, and Spearman’s rank correlation coefficient (Rs) to measure the strength of associations between them, as the distribution of quantitative variables differed from the normal distribution in most cases. To compare qualitative variables between groups we used Fisher’s exact test. Polymorphisms in genes previously linked with AMD including CFH His402Tyr and ARMS2 Ala69Ser were analyzed. Associations between genotype and plasma miRNAs expression were analyzed with Kruskal–Wallis test comparing three genotypes followed by Mann–Whitney test comparing TT + TC vs. CC genotypes (dominant model) and TT vs. TC + CC (recessive model). Multivariate analysis of AMD as an independent variable associated with concentrations of angiogenesis-regulating factors and miRNAs was performed using a general linear model (GLM) adjusted for age, sex, and smoking status (pack-years), with logarithmic transformation applied to the dependent variables to normalize their distributions. *p* < 0.05 was considered statistically significant. In genotype-phenotype association analysis Bonferroni correction for multiple tests was applied: since 14 mRNAs were analyzed in two genetic models, the corrected *p*-value threshold level for Mann–Whitney test was 0.05/14/2 = 0.0018.

## Figures and Tables

**Table 1:** Characteristics of the study groups.

Parameter	AMD Group	Control Group	*p*-Value *
Number of subjects	354	121	—
Sex (male/female)	135/219	32/89	**0.02**
Patient’s age (years) (mean ± SD)	73.4 ± 8.0	73.1 ± 6.0	0.41
BMI (kg/m^2^) (mean ± SD)	26.93 ± 4.22	26.56 ± 3.66	0.43
WHR (arbitrary units) (mean ± SD)	0.90 ± 0.09	0.88 ± 0.09	0.13
Waist circumference (cm) (mean ± SD)	103.25 ± 9.09	102.10 ± 7.26	0.33
MAP (mmHg) (mean ± SD)	98.30 ± 11.10	98.72 ± 9.66	0.86
Current smokers (%)	13.62%	6.25%	0.0503
Former smokers (%)	51.39%	30.93%	**0.0004**
Smoking pack-years (mean ± SD)	13.58 ± 18.91	6.00 ± 13.09	**0.00007**
Period without smoking (years) (mean ± SD)	6.79 ± 10.90	5.30 ± 10.23	0.055
Iris color (dark/light)	91/261	26/95	0.39
Outdoor/indoor working conditions	40.11/59.89%	33.06/66.94%	0.19
Hypertension (%)	64.71%	71.13%	0.27
Duration of hypertension (years) (mean ± SD)	8.17 ± 9.45	9.15 ± 9.86	0.27
History of ischemic heart disease (%)	16.15%	11.34%	0.33
Duration of ischemic heart disease (years) (mean ± SD)	1.23 ± 4.17	0.81 ± 3.28	0.26
History of cardiac infarction (%)	6.21%	6.19%	1.00
History of cerebral stroke (%)	2.81%	3.09%	1.00
History of peripheral artery disease (%)	4.97%	6.19%	0.61
History of aortic aneurysm (%)	1.57%	0.00%	0.59
Hypotensive drugs/vasodilators	65.02%	70.10%	0.39
Hormonal drugs	17.13%	20.62%	0.45
Thyroxine	13.71%	20.62%	0.11
Steroids	1.87%	1.03%	1.00
Other hormonal drugs	1.25%	0.00%	0.58
Statins	26.63%	36.08%	0.07
NSAIDs	20.19%	19.59%	1.00
Cardiac medications/antiarrhythmic drugs	13.93%	14.43%	0.87
Antiasthmatic drugs	7.43%	3.09%	0.16
Antidepressants	4.66%	5.15%	0.79

* Mann–Whitney test/Fisher’s exact test. In bold, *p*-value < 0.05, which was considered statistically significant.

**Table:** (**a**)

	AMD Group		Control Group		*p*-Value *
	N	Mean ± SD	N	Mean ± SD	
Angiogenin (pg/mL)	312	3802.28 ± 2396.12	119	5341.18 ± 3800.49	**<0.001**
Angiopoietin-1	313	7371.09 ± 4735.69	119	8032.73 ± 5305.49	0.338
Endostatin (pg/mL)	313	34,804.05 ± 11,909.86	119	40,891.63 ± 13,835.72	**<0.001**
FGF-basic (pg/mL)	313	162.13 ± 72.68	118	188.32 ± 86.49	**0.002**
FGF-acidic (pg/mL)	313	98.53 ± 65.14	119	86.52 ± 38.86	0.117
PDGF-AA (pg/mL)	313	623.55 ± 392.73	119	559.00 ± 328.33	0.261
PlGF (pg/mL)	313	10.45 ± 4.32	119	13.30 ± 6.86	**<0.001**
Thrombospondin-2 (pg/mL)	313	9557.33 ± 5308.18	119	8781.31 ± 4659.82	0.201
VEGF (pg/mL)	313	37.94 ± 22.72	119	46.69 ± 34.89	0.137
VEGF-D (pg/mL)	313	189.35 ± 81.74	118	196.68 ± 94.95	0.870

N: number of observations; * Mann–Whitney test. In bold, *p*-value < 0.05, which was considered statistically significant.

**Table:** (**b**)

	Dry AMD Group		Wet AMD Group		*p*-Value *
	N	Mean ± SD	N	Mean ± SD	
Angiogenin (pg/mL)	159	3908.10 ± 2493.55	153	3692.32 ± 2293.48	0.553
Angiopoietin-1	160	7060.17 ± 4163.04	153	7696.25 ± 5262.75	0.622
Endostatin (pg/mL)	160	34,307.44 ± 11,932.47	153	35,323.38 ± 11,903.06	0.393
FGF-basic (pg/mL)	160	164.67 ± 62.95	153	159.48 ± 81.76	0.128
FGF-acidic (pg/mL)	160	101.54 ± 59.75	153	95.39 ± 70.40	0.241
PDGF-AA (pg/mL)	160	624.48 ± 384.43	153	622.59 ± 402.48	0.859
PlGF (pg/mL)	160	10.47 ± 3.53	153	10.44 ± 5.04	0.202
Thrombospondin-2 (pg/mL)	160	9614.22 ± 5704.43	153	9497.83 ± 4877.54	0.747
VEGF (pg/mL)	160	39.49 ± 24.27	153	36.32 ± 20.92	0.184
VEGF-D (pg/mL)	160	190.05 ± 73.08	153	188.62 ± 90.14	0.359

N: number of observations; * Mann–Whitney test. In bold, *p*-value < 0.05, which was considered statistically significant.

**Table:** (**a**)

	AMD Group		Control Group		*p*-Value *
	N	Mean ± SD	N	Mean ± SD	
miRNA-9-5p	331	2.078 ± 3.050	113	1.900 ± 0.879	0.128
miRNA-16-5p	334	16.370 ± 12.218	114	10.159 ± 10.085	**<0.001**
miRNA-17-3p	332	5.369 ± 3.554	114	4.428 ± 3.292	**<0.001**
miRNA-17-5p	333	2.600 ± 2.498	113	1.560 ± 0.888	**<0.001**
miRNA-21-3p	334	1.772 ± 1.458	113	2.408 ± 2.224	**<0.001**
miRNA-23a-3p	333	2.005 ± 2.6775	113	1.611 ± 2.080	**<0.001**
miRNA-30b	332	1.274 ± 0.606	113	1.306 ± 0.900	0.383
miRNA-93	331	4.195 ± 3.508	114	3.882 ± 2.992	0.860
miRNA-126-5p	331	1.262 ± 0.400	113	1.020 ± 0.290	**<0.001**
miRNA-146a	332	0.712 ± 0.587	113	0.692 ± 0.825	**0.002**
miRNA-150-5p	335	1.716 ± 6.580	114	3.318 ± 12.830	0.265
miRNA-155-5p	334	1.551 ± 5.548	113	1.903 ± 2.224	**<0.001**
miRNA-191-5p	333	0.963 ± 0.685	113	1.178 ± 1.037	**0.021**
miRNA-223-3p	333	1.813 ± 6.424	113	1.691 ± 4.181	**0.019**

N: number of observations; * Mann–Whitney test; Expression was calculated in relation to the expression of miR-93 as reference miRNA. In bold, *p*-value < 0.05, which was considered statistically significant.

**Table:** (**b**)

	Dry AMD Group		Wet AMD Group		*p*-Value *
	N	Mean ± SD	N	Mean ± SD	
miRNA-9-5p	164	2.344 ± 4.085	167	1.818 ± 1.405	0.426
miRNA-16-5p	165	14.524 ± 11.308	169	18.172 ± 12.823	**0.002**
miRNA-17-3p	165	5.082 ± 3.182	167	5.654 ± 3.875	0.292
miRNA-17-5p	165	2.659 ± 2.544	168	2.542 ± 2.458	0.839
miRNA-21-3p	165	1.851 ± 1.727	169	1.696 ± 1.135	0.627
miRNA-23a-3p	165	2.105 ± 3.176	168	1.908 ± 2.080	**0.040**
miRNA-30b	165	1.174 ± 0.598	167	1.373 ± 0.600	**<0.001**
miRNA-93	164	3.798 ± 3.147	167	4.585 ± 3.802	0.099
miRNA-126-5p	164	1.278 ± 0.336	167	1.247 ± 0.455	0.079
miRNA-146a	165	0.702 ± 0.530	167	0.722 ± 0.640	0.922
miRNA-150-5p	165	1.569 ± 4.142	170	1.859 ± 8.299	0.836
miRNA-155-5p	165	1.233 ± 0.774	169	1.862 ± 7.761	0.431
miRNA-191-5p	165	0.877 ± 0.636	168	1.048 ± 0.721	**0.026**
miRNA-223-3p	164	1.587 ± 4.228	169	2.033 ± 8.008	0.291

N: number of observations; * Mann–Whitney test; Expression was calculated in relation to the expression of miR-93 as reference miRNA. In bold, *p*-value < 0.05, which was considered statistically significant.

**Table 4:** Spearman’s correlation coefficients for plasma miRNAs and angiogenesis-regulating factors in all AMD patients (both wet and dry AMD subtypes included).

miRNA	Angiogenin	Angiopoietin-1	Endostatin	FGF-Basic	FGF-Acidic	PDGF-AA	PlGF	Thrombospondin-2	VEGF	VEGF_D
**miRNA-9-5p**	0.023	0.011	−0.020	0.044	−0.008	−0.022	0.019	−0.005	−0.036	0.038
**miRNA-16-5p**	−0.031	−0.011	0.015	−0.037	−0.082	0.098	−0.070	−0.038	0.019	−0.075
**miRNA-17-3p**	0.098	−0.094	0.012	−0.053	**−0.175**	−0.069	**−0.147**	**−0.129**	−0.072	**−0.155**
**miRNA-17-5p**	**0.247**	0.031	**0.209**	0.102	**−0.214**	−0.054	0.051	−0.015	−0.073	−0.083
**miRNA-21-3p**	0.014	0.057	-0.019	0.017	−0.002	0.019	**0.114**	−0.038	0.082	0.060
**miRNA-23a-3p**	**−0.239**	0.062	**−0.194**	−0.066	**0.254**	**0.148**	−0.027	0.054	**0.125**	0.111
**miRNA-30b**	**−0.155**	0.047	−0.068	−0.015	0.073	**0.138**	−0.101	−0.034	0.113	−0.030
**miRNA-93**	**0.218**	0.057	**0.192**	0.066	**−0.237**	−0.020	0.049	0.013	**−0.137**	−0.085
**miRNA-126-5p**	0.054	0.045	0.108	−0.013	−0.096	0.045	0.014	−0.030	−0.006	−0.071
**miRNA-146a**	**−0.194**	0.071	**−0.208**	−0.020	**0.245**	0.084	−0.035	0.041	0.093	0.016
**miRNA-150-5p**	**0.230**	**0.140**	**0.211**	0.076	**−0.231**	−0.064	**0.161**	0.030	−0.087	−0.016
**miRNA-155-5p**	**−0.312**	−0.001	**−0.246**	−0.073	**0.217**	**0.155**	**−0.159**	0.067	**0.139**	0.073
**miRNA-191-5p**	**0.134**	−0.034	0.111	0.028	−0.057	−0.062	0.057	−0.010	−0.087	−0.005
**miRNA-223-3p**	−0.310	0.071	**−0.225**	**−0.135**	**0.262**	**0.179**	**−0.166**	−0.069	**0.169**	0.068

Statistically significant results (*p* < 0.05) are shown in bold. The grey background corresponds to unique correlations that were statistically significant only in the AMD group and not in controls.

**Table 5:** Spearman’s correlation coefficients for plasma miRNAs and angiogenesis-regulating factors in controls.

miRNA	Angiogenin	Angiopoietin-1	Endostatin	FGF-Basic	FGF-Acidic	PDGF-AA	PlGF	Thrombospondin-2	VEGF	VEGF_D
**miRNA-9-5p**	0.114	0.004	0.153	0.010	−0.027	0.010	−0.186	−0.179	−0.029	0.073
**miRNA-16-5p**	−0.145	−0.035	0.008	0.122	0.114	−0.026	−0.062	−0.019	0.106	0.040
**miRNA-17-3p**	−0.046	−0.088	0.051	−0.004	0.026	−0.032	−0.159	−0.083	0.039	0.001
**miRNA-17-5p**	**0.225**	−0.018	0.162	−0.082	0.010	0.098	−0.028	−0.168	0.079	0.019
**miRNA-21-3p**	0.010	0.014	0.032	−0.047	−0.145	−0.031	−0.089	−0.099	−0.114	−0.129
**miRNA-23a-3p**	−0.035	**−0.220**	0.038	**−0.188**	−0.042	−0.134	−0.024	**−0.193**	−0.158	0.009
**miRNA-30b**	0.151	−0.139	0.115	−0.132	0.035	0.008	−0.052	**−0.211**	0.043	−0.036
**miRNA-93**	−0.007	−0.111	0.036	−0.022	−0.096	−0.053	−0.063	−0.114	−0.145	−0.033
**miRNA-126-5p**	**−0.228**	0.031	−0.090	0.092	0.107	0.011	0.172	0.064	0.042	0.042
**miRNA-146a**	−0.131	**−0.228**	−0.106	−0.117	−0.031	−0.129	0.037	−0.155	−0.150	−0.010
**miRNA-150-5p**	−0.131	−0.070	0.004	0.049	0.052	−0.071	−0.155	−0.094	0.052	0.045
**miRNA-155-5p**	−0.046	0.026	−0.138	0.114	0.072	0.005	**0.216**	−0.013	0.015	0.138
**miRNA-191-5p**	0.027	−0.037	0.052	−0.049	0.016	0.106	0.026	−0.082	0.025	−0.009
**miRNA-223-3p**	**−0.210**	−0.081	0.001	0.152	0.064	−0.160	0.003	−0.088	−0.042	0.007

Statistically significant results (*p* < 0.05) are shown in bold.

**Table 6:** Plasma miRNA expression profiles in patients with different alleles in CFH Y402H.

	*p*-Value ^1^	TT		TC + CC		*p*-Value ^2^	CC		TT + TC		*p*-Value ^2^
		Mean	Median	Mean	Median		Mean	Median	Mean	Median	
**miRNA-9-5p**	0.776	2.225	1.533	1.931	1.672	0.927	1.910	1.583	2.036	1.734	0.535
**miRNA-16-5p**	**0.002**	20.079	16.305	15.004	12.011	0.0007 *	16.187	12.252	15.981	12.537	0.567
**miRNA-17-3p**	0.101	5.805	5.290	5.056	4.460	**0.049**	5.146	4.695	5.247	4.490	0.960
**miRNA-17-5p**	**0.012**	3.333	2.098	2.409	1.527	**0.005**	2.139	1.507	2.844	1.677	0.058
**miRNA-21-3p**	0.502	1.916	1.283	1.731	1.286	0.654	1.627	1.248	1.845	1.324	0.241
**miRNA-23a-3p**	**0.026**	1.447	1.123	2.119	1.325	0.057	1.607	1.285	2.176	1.302	0.288
**miRNA-30b**	0.119	1.340	1.196	1.236	1.103	**0.043**	1.243	1.094	1.266	1.157	0.265
**miRNA-93**	0.213	4.852	3.891	4.015	2.691	0.109	3.876	2.434	4.357	3.199	0.204
**miRNA-126-5p**	0.562	1.273	1.221	1.241	1.179	0.712	1.204	1.167	1.271	1.191	0.283
**miRNA-146a**	0.235	0.613	0.474	0.727	0.602	0.230	0.674	0.506	0.719	0.604	0.499
**miRNA-150-5p**	0.383	1.232	0.744	1.808	0.667	0.246	2.414	0.656	1.305	0.697	0.256
**miRNA-155-5p**	0.432	1.091	0.948	1.760	1.080	0.195	2.375	1.154	1.227	0.994	0.648
**miRNA-191-5p**	0.628	0.955	0.883	0.953	0.863	0.917	0.914	0.789	0.975	0.899	0.353
**miRNA-223-3p**	0.056	2.919	0.742	1.520	1.022	**0.045**	1.839	1.009	1.786	1.021	0.628

Statistically significant results (*p* < 0.05) are shown in bold. ^1^ Kruskal-Wallis test, ^2^ Mann-Whitney test, * statistically significant when correction for multiple testing was applied (*p* < 0.0018).

## Abbreviations

AMD	Age-related macular degeneration
ANGPT	Angiopoietin
CNV	Choroidal neovascularization
EC	Endothelial cells
FGF	Fibroblast growth factor
MAP	Mean arterial pressure
MIP	Molecular inversion probes
MMP	Matrix metalloproteinase
NSAID	Non-steroidal anti-inflammatory drugs
OCT	Optical coherence tomography
OIR	Oxygen-induced retinopathy
PDGF	Platelet-derived growth factor
PEDF	Pigment epithelium-derived growth factor
RPE	Retinal pigment epithelium
SD	Standard deviation
UMI	Unique molecular identifiers sequences
VEGF	Vascular endothelial growth factor
WHR	Waist–hip ratio
